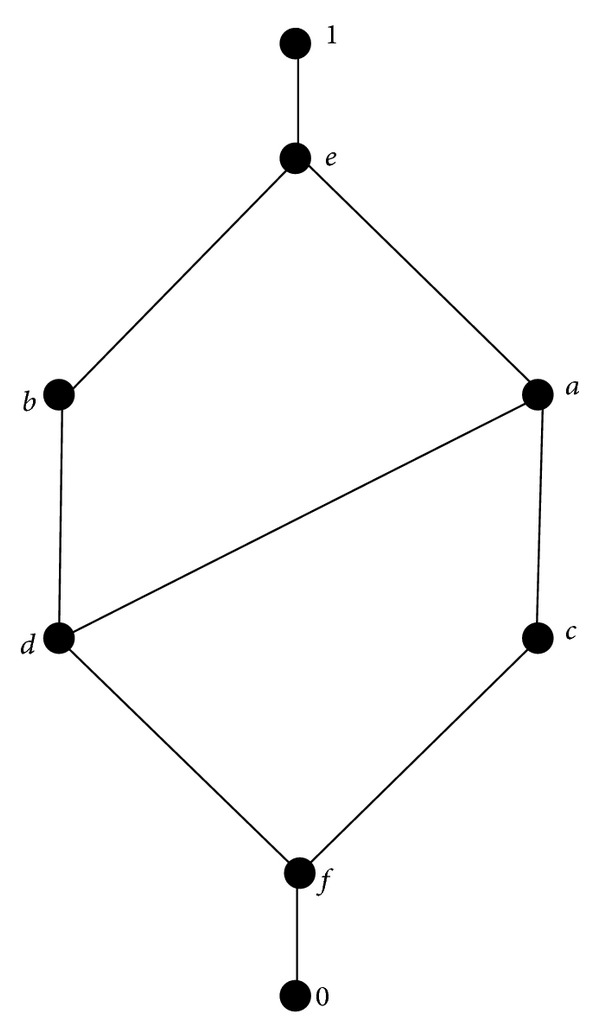# The Axioms Independence of Pseudo-Weak-*R*_0_ Algebras and Filters

**DOI:** 10.1155/2014/854168

**Published:** 2014-07-07

**Authors:** Yong Lin Liu

**Affiliations:** Department of Mathematics and Computer, Wuyi University, Wuyishan, Fujian 354300, China

## Abstract

The most simplified axiom systems of pseudo-weak-*R*_0_ algebras and pseudo-*R*_0_ algebras are obtained, and the mutually independence of axioms is proved. We introduce the notions of filters and normal filters in pseudo-weak-*R*_0_ algebras. The structures and properties of the generated filters and generated normal filters in pseudo-weak-*R*_0_ algebras are obtained. These can be seen as noncommutative generalizations of the corresponding ones in weak-*R*_0_ algebras.

## 1. Introduction

In recent years, the study of logic algebras and their noncommutative generalization—pseudo-logic algebras—has become of greater focus in the field of logic. BCK and BCI algebras were introduced by Imai and Iseki [1] and have been extensively investigated by many researchers. Georgescu and Iorgulescu [2] introduced the notion of a pseudo-BCK algebra as a noncommutative generalization of a BCK-algebra. Liu et al. [3] investigated the theory of pseudo-BCK algebras. MV-algebras were introduced by Chang in [4] as an algebraic tool to study the infinitely valued logic of Lukasiewicz. Georgescu and Iorgulescu [5] introduced pseudo MV-algebras which is a noncommutative generalization of MV-algebras. The notion of BL-algebras was introduced by Hajek [6] as the algebraic structures for his Basic Logic. Georgescu and Iorgulescu [7] introduced the notion of pseudo-BL algebras by dropping commutative axioms in BL-algebras. di Nola et al. [8, 9], Zhang and Fan [10], and Zhan et al. [11] investigated in detail the theory of pseudo-BL algebras. MTL-algebras [12] are the algebraic structures for Esteva-Godo monoidal *t*-norm based logic, many-valued propositional calculus that formalizes the structure of the real unit interval [0,1], induced by a left-continuous *t*-norm. Flondor et al. [13] presented pseudo-MTL algebras as a noncommutative generalization of MTL-algebras. IMTL-algebras [12] are the algebraic counterpart for involutive monoidal *t*-norm logic, an extension of MTL-algebras. NM-algebras [12] are the algebraic counterpart for nilpotent minimum logic, an extension of IMTL-algebras. Iorgulescu [14] and Liu and zhang [15] introduced and studied the pseudo-IMTL algebras and pseudo-NM algebras. *R*_0_ algebras were introduced by Wang [16] as the algebraic structure for his formal deductive system L* of fuzzy propositional calculus. Weak-*R*_0_ algebras [16] are the generalization of *R*_0_ algebras. The research on *R*_0_ algebras has attracted more and more attention [17].

In [18], we introduced and studied the pseudo-weak-*R*_0_ algebras and pseudo-*R*_0_ algebras. They are noncommutative generalizations of the weak-*R*_0_ algebras and *R*_0_ algebras, respectively. Some properties, the noncommutative forms of the properties in weak-*R*_0_ algebras and *R*_0_ algebras, were investigated. We showed that pseudo-weak-*R*_0_ algebras are categorically isomorphic to pseudo-IMTL algebras, and pseudo-*R*_0_ algebras are categorically isomorphic to pseudo-NM algebras.

Based on these results, in this paper, our study focused on the axioms independence and filter theory in pseudo-weak-*R*_0_ algebras and pseudo-*R*_0_ algebras. The most simplified axiom systems of pseudo-weak-*R*_0_ algebras and pseudo-*R*_0_ algebras are obtained, and the mutually independence of axioms is proved. The notions of filters and normal filters in pseudo-weak-*R*_0_ algebras are introduced. The structures and properties of the generated filters and generated normal filters in pseudo-weak-*R*_0_ algebras are obtained. These can be seen as noncommutative generalizations of the corresponding ones in weak-*R*_0_ algebras.

## 2. Preliminaries

We recall some definitions and results which will be used in the sequel.

**Definition 1 (see [12]).**

An IMTL (involutive MTL) algebra is a structure (*A*, ∨, ∧, ⊙, →, 0,1) of type (2,2, 2,2, 0,0) such that for all *x*, *y*, *z* ∈ *A*:

(B1) (*A*, ∨, ∧, 0,1) is a bounded lattice,
(B2) (*A*, ⊙, 1) is a monoid,
(B3) *x*⊙*y* ≤ *z* if and only if *x* ≤ *y* → *z*,
(B4) (*x* → *y*)∨(*y* → *x*) = 1,
(B5) *x* ^−−^ = *x*,

where *x* ^−^ = *x* → 0.

An NM (nilpotent minimum) algebra is an IMTL algebra satisfying the following condition:

(B6) (*x*⊙*y*)^−^∨((*x*∧*y*)→(*x*⊙*y*)) = 1.

**Definition 2 (see [14, 15]).**

A pseudo-IMTL (pseudo-involutive MTL) algebra is a structure (*A*, ∨, ∧, ⊙, →, ⇝, 0,1) of type (2,2, 2,2, 2,0, 0) such that for all *x*, *y*, *z* ∈ *A*:

(pB1) (*A*, ∨, ∧, 0,1) is a bounded lattice,
(pB2) (*A*, ⊙, 1) is a monoid,
(pB3) *x*⊙*y* ≤ *z* if and only if *x* ≤ *y* → *z* if and only if *y* ≤ *x*⇝*z*,
(pB4) (*x* → *y*)∨(*y* → *x*) = (*x*⇝*y*)∨(*y*⇝*x*) = 1,
(pB5) *x* ^~−^ = *x* ^−~^ = *x*,

where *x* ^−^ = *x* → 0 and *x* ^~^ = *x*⇝0.

A pseudo-NM (pseudo-nilpotent minimum) algebra is a pseudo-IMTL algebra satisfying the following condition:

(pB6) (*x*⊙*y*)^−^∨((*x*∧*y*)→(*x*⊙*y*)) = (*x*⊙*y*)^~^∨((*x*∧*y*)⇝(*x*⊙*y*)) = 1.

**Definition 3 (see [16, 19]).**

Let *M* be a (¬, ∧, ∨, →)-type algebra, where ¬ is a unary operation and ∧, ∨, and → are binary operations. If there is a partial ordering ≤ on *M*, such that (*M*, ≤) is a bounded distributive lattice, ∧ and ∨ are infimum and supremum operations with respect to ≤, ¬ is an order-reversing involution with respect to ≤, and the following conditions hold for any *a*, *b*, *c* ∈ *M*

(R1) ¬*a* → ¬*b* = *b* → *a*,
(R2) 1 → *a* = *a*, *a* → *a* = 1,
(R3) *b* → *c* ≤ (*a* → *b*)→(*a* → *c*),
(R4) *a* → (*b* → *c*) = *b* → (*a* → *c*),
(R5) *a* → (*b*∨*c*) = (*a* → *b*)∨(*a* → *c*), *a* → (*b*∧*c*) = (*a* → *b*)∧(*a* → *c*),

where 1 is the largest element of *M*, and then we call *M* a weak-*R*_0_ algebra.

An *R*_0_ algebra *M* is a weak-*R*_0_ algebra satisfying the additional condition as follows:

(R6) (*a* → *b*)∨((*a* → *b*)→(¬*a*∨*b*)) = 1.

**Definition 4 (see [18]).**

A pseudo-weak-*R*_0_ algebra is a structure(*A*, ∧, ∨, →, ⇝,^−^,^~^, 0,1) such that (*A*, ∧, ∨, 0,1) is a bounded distributive lattice, ^−^ and ^~^ are order-reversing pseudo-involution (i.e., if *x* ≤ *y*, then *y* ^−^ ≤ *x* ^−^ and *y* ^~^ ≤ *x* ^~^; *x* ^~−^ = *x* ^−~^ = *x*), and the following axioms hold for any *x*, *y*, *z* ∈ *A*:

(pR1) *x* → *y* = *y* ^−^⇝*x* ^−^, *x*⇝*y* = *y* ^~^ → *x* ^~^,
(pR2∗) 1 → *x* = 1⇝*x* = *x*; *x* → *x* = *x*⇝*x* = 1,
(pR3) *x* → *y* ≤ (*z* → *x*)→(*z* → *y*), *x*⇝*y* ≤ (*z*⇝*x*)⇝(*z*⇝*y*),
(pR4) *x* → (*y*⇝*z*) = *y*⇝(*x* → *z*),
(pR5∗) *x* → (*y*∨*z*) = (*x* → *y*)∨(*x* → *z*), *x*⇝(*y*∨*z*) = (*x*⇝*y*)∨(*x*⇝*z*); *x* → (*y*∧*z*) = (*x* → *y*)∧(*x* → *z*), *x*⇝(*y*∧*z*) = (*x*⇝*y*)∧(*x*⇝*z*).

A pseudo-*R*_0_ algebra *A* is a pseudo-weak-*R*_0_ algebra satisfying the additional axiom as follows:

(pR6) (*x* → *y*)∨((*x* → *y*)⇝(*x* ^−^∨*y*)) = (*x*⇝*y*)∨((*x*⇝*y*)→(*x* ^~^∨*y*)) = 1.

In [18], we also have another simplified definition.

**Definition 5 (see [18]).**

A pseudo-weak-*R*_0_ algebra is a structure (*A*, ∧, ∨, →, ⇝,^−^,^~^, 0,1) satisfying

(pL1) (*A*, ∧, ∨, 0,1) is a bounded lattice,
(pL2) if *x* ≤ *y*, then *y* ^−^ ≤ *x* ^−^ and *y* ^~^ ≤ *x* ^~^,
(pL3) *x* ^~−^ = *x* ^−~^ = *x*,
(pR1) *x* → *y* = *y* ^−^⇝*x* ^−^, *x*⇝*y* = *y* ^~^ → *x* ^~^,
(pR2) 1 → *x* = 1⇝*x* = *x*,
(pR3) *x* → *y* ≤ (*z* → *x*)→(*z* → *y*), *x*⇝*y* ≤ (*z*⇝*x*)⇝(*z*⇝*y*),
(pR4) *x* → (*y*⇝*z*) = *y*⇝(*x* → *z*),
(pR5) *x* → (*y*∨*z*) = (*x* → *y*)∨(*x* → *z*), *x*⇝(*y*∨*z*) = (*x*⇝*y*)∨(*x*⇝*z*).

A pseudo-*R*_0_ algebra *A* is a pseudo-weak-*R*_0_ algebra satisfying the additional axiom as follows:

(pR6) (*x* → *y*)∨((*x* → *y*)⇝(*x* ^−^∨*y*)) = (*x*⇝*y*)∨((*x*⇝*y*)→(*x* ^~^∨*y*)) = 1.

**Proposition 6 (see [18]).**

*In a pseudo-weak-R*_0_ *algebra, the following properties hold:*

1. 0^~^ = 0^−^ = 1, 1^~^ = 1^−^ = 0,
2. *x* ^−^ = *x* → 0, *x* ^~^ = *x*⇝0,
3. *x*⇝*x* = *x* → *x* = 1,
4. *x* ≤ *y if and only if x*⇝*y* = 1* if and only if x* → *y* = 1,
5. (⋀_*i*∈*I*_ *x*_*i*_)^~^ = ⋁_*i*∈*I*_ *x*_*i*_^~^, (⋀_*i*∈*I*_ *x*_*i*_)^−^ = ⋁_*i*∈*I*_ *x*_*i*_^−^ *, whenever the arbitrary meets and unions exist,*
6. (⋁_*i*∈*I*_ *x*_*i*_)^~^ = ⋀_*i*∈*I*_ *x*_*i*_^~^, (⋁_*i*∈*I*_ *x*_*i*_)^−^ = ⋀_*i*∈*I*_ *x*_*i*_^−^ *, whenever the arbitrary meets and unions exist,*
7. * if x* ≤ *y, then z*⇝*x* ≤ *z*⇝*y and z* → *x* ≤ *z* → *y*,
8. * if x* ≤ *y, then y*⇝*z* ≤ *x*⇝*z and y* → *z* ≤ *x* → *z*,
9. (*x*∧*y*)⇝*z* = (*x*⇝*z*)∨(*y*⇝*z*), (*x*∧*y*) → *z* = (*x* → *z*)∨(*y* → *z*),
10. *x*⇝(*y*∧*z*) = (*x*⇝*y*)∧(*x*⇝*z*), *x* → (*y*∧*z*) = (*x* → *y*)∧(*x* → *z*),
11. (*x*∨*y*)⇝*z* = (*x*⇝*z*)∧(*y*⇝*z*), (*x*∨*y*) → *z* = (*x* → *z*)∧(*y* → *z*),
12. *x* ^~^∨*y* ≤ *x*⇝*y*, *x* ^−^∨*y* ≤ *x* → *y*,
13. *x*⇝*y* ≤ (*y*⇝*z*)→(*x*⇝*z*), *x* → *y* ≤ (*y* → *z*)⇝(*x* → *z*),
14. (*A*, ∧, ∨, 0,1)* is a bounded distributive lattice,*
15. *x*⇝*y* ≤ *x*∨*z*⇝*y*∨*z*, *x* → *y* ≤ *x*∨*z* → *y*∨*z*,
16. *x*⇝*y* ≤ *x*∧*z*⇝*y*∧*z*, *x* → *y* ≤ *x*∧*z* → *y*∧*z*,
17. (*x*⇝*y*)≤(*x*⇝*z*)∨(*z*⇝*y*), (*x* → *y*)≤(*x* → *z*)∨(*z* → *y*),
18. (*x*⇝*y*)∨(*y*⇝*x*) = (*x* → *y*)∨(*y* → *x*) = 1,
19. *x* ≤ (*x* → *y*)⇝*y*, *x* ≤ (*x*⇝*y*) → *y*,
20. *x* → *y* = ((*x* → *y*)⇝*y*) → *y*, *x*⇝*y* = ((*x*⇝*y*) → *y*)⇝*y*,
21. *x* → (*y* → *x*) = *x*⇝(*y*⇝*x*) = *x*⇝(*y* → *x*) = *x* → (*y*⇝*x*) = 1,
22. *x* ^−^ → (*x* → *y*) = *x* ^~^⇝(*x*⇝*y*) = *x* ^−^⇝(*x* → *y*) = *x* ^~^ → (*x*⇝*y*) = 1,
23. *y* ≤ (*x*⇝*y*)∧(*x* → *y*),
24. *x*∨*y* = ((*x*⇝*y*) → *y*)∧((*y*⇝*x*) → *x*) = ((*x* → *y*)⇝*y*)∧((*y* → *x*)⇝*x*),
25. (*x*∨*y*) → *x* = *y* → *x*, (*x*∨*y*)⇝*x* = *y*⇝*x*,
26. *x* → (*x*∧*y*) = *x* → *y*, *x*⇝(*x*∧*y*) = *x*⇝*y*,
27. *x* ≤ *y* ^−^ *if and only if y* ≤ *x* ^~^,
28. *x* → *y* ^~^ = *y*⇝*x* ^−^, *x*⇝*y* ^−^ = *y* → *x* ^~^,
29. (*x* → *y* ^−^)^~^ = (*y*⇝*x* ^~^)^−^.

*In a pseudo-weak-R*_0_ *algebra (pseudo-R*_0_ *algebra) A* *, we define a binary operation *⊙* as follows, for any x*, *y* ∈ *A:*

(30) (1) $$\begin{array}{c} x⊙y={\left(x⟶y^{-}\right)}^{\sim }={\left(y⇝x^{\sim }\right)}^{-}. \end{array}$$

**Proposition 7 (see [18]).**

*In a pseudo-weak-R*_0_ *algebra, the following properties hold:*

(31) *x* → *y* = (*x*⊙*y* ^~^)^−^, *x*⇝*y* = (*y* ^−^⊙*x*)^~^,
(32) (*x*⊙*y*)⊙*z* = *x*⊙(*y*⊙*z*),
(33) 1⊙*x* = *x*⊙1 = *x*,
(34) *x*⊙*y* ≤ *z* *if and only if x* ≤ *y* → *z* *if and only if y* ≤ *x*⇝*z*,
(35) *x*⊙(*x*⇝*y*) ≤ *y* ≤ *x*⇝(*x*⊙*y*), (*x* → *y*)⊙*x* ≤ *y* ≤ *x* → (*y*⊙*x*),
(36) *x*⊙(*x*⇝*y*) ≤ *x* ≤ *y*⇝(*y*⊙*x*), (*x* → *y*)⊙*x* ≤ *x* ≤ *y* → (*x*⊙*y*),
(37) * if x* ≤ *y, then x*⊙*z* ≤ *y*⊙*z and z*⊙*x* ≤ *z*⊙*y*,
(38) *x*⊙(*x*⇝*y*) ≤ *x*∧*y*, (*x* → *y*)⊙*x* ≤ *x*∧*y*,
(39) *x*⊙0 = 0⊙*x* = 0,
(40) *x*⊙(⋁_*i*∈*I*_ *x*_*i*_) = ⋁_*i*∈*I*_(*x*⊙*x*_*i*_), (⋁_*i*∈*I*_ *x*_*i*_)⊙*x* = ⋁_*i*∈*I*_(*x*_*i*_⊙*x*)*, whenever the arbitrary unions exist,*
(41) (*x*⊙*y*) → *z* = *x* → (*y* → *z*), (*y*⊙*x*)⇝*z* = *x*⇝(*y*⇝*z*),
(42) *y*⇝(⋀_*i*∈*I*_ *x*_*i*_) = ⋀_*i*∈*I*_(*y*⇝*x*_*i*_), *y* → (⋀_*i*∈*I*_ *x*_*i*_) = ⋀_*i*∈*I*_(*y* → *x*_*i*_)*, whenever the arbitrary meets exist,*
(43) (⋁_*i*∈*I*_ *x*_*i*_)⇝*y* = ⋀_*i*∈*I*_(*x*_*i*_⇝*y*), (⋁_*i*∈*I*_ *x*_*i*_) → *y* = ⋀_*i*∈*I*_(*x*_*i*_ → *y*)*, whenever the arbitrary unions and meets exist,*
(44) *x*⊙*x* ^~^ = *x* ^−^⊙*x* = 0,
(45) *x*⊙*y* ≤ *x*∧*y* ≤ *x*, *y*,
(46) *x*∨(*y*⊙*z*)≥(*x*∨*y*)⊙(*x*∨*z*),
(47) *x* → *y* ≤ (*x*⊙*z*)→(*y*⊙*z*), *x*⇝*y* ≤ (*z*⊙*x*)⇝(*z*⊙*y*),
(48) *x*⊙(*y* → *z*) ≤ *y* → (*x*⊙*z*), (*y*⇝*z*)⊙*x* ≤ *y*⇝(*z*⊙*x*).

## 3. The Axioms Independence of Pseudo-Weak-*R*_0_ Algebras

We investigate the axioms independence of pseudo-*R*_0_ algebras and pseudo-weak-*R*_0_ algebras. Hence, we obtain most simplified axiom systems of pseudo-weak-*R*_0_ algebras and pseudo-*R*_0_ algebras.

**Theorem 8 .**

A structure (*A*, ∨, ∧, →, ⇝,^−^,^~^, 0,1) is a pseudo-weak-*R*_0_ algebra if and only if it satisfies the following conditions:

(pL1) (*A*, ∧, ∨, 0,1) is a bounded lattice,
(pL3′) 1^~−^ = 1^−~^ = 1, 0^~−^ = 0^−~^ = 0,
(pR1) *x* → *y* = *y* ^−^⇝*x* ^−^, *x*⇝*y* = *y* ^~^ → *x* ^~^,
(pR2) 1 → *x* = 1⇝*x* = *x*,
(pR3) *x* → *y* ≤ (*z* → *x*)→(*z* → *y*), *x*⇝*y* ≤ (*z*⇝*x*)⇝(*z*⇝*y*),
(pR5) *x* → (*y*∨*z*) = (*x* → *y*)∨(*x* → *z*), *x*⇝(*y*∨*z*) = (*x*⇝*y*)∨(*x*⇝*z*).

**Proof**

Necessity is obvious. For sufficiency, it only needs to show axioms (pL2), (pL3), and (pR4) of Definition 5 hold. We first show the following three properties hold:

- *x* → *y* ≤ (*y* → *z*)⇝(*x* → *z*), *x*⇝*y* ≤ (*y*⇝*z*)→(*x*⇝*z*),
- *x* → *x* = *x*⇝*x* = 1,
- *x* ≤ *y* if and only if *x* → *y* = 1 if and only if *x*⇝*y* = 1.

In fact, by (pR1) and (pR3), we have *x* → *y* = *y* ^−^⇝*x* ^−^ ≤ (*z* ^−^⇝*y* ^−^)⇝(*z* ^−^⇝*x* ^−^) = (*y* → *z*)⇝(*x* → *z*), *x*⇝*y* = *y* ^~^ → *x* ^~^ ≤ (*z* ^~^ → *y* ^~^)→(*z* ^~^ → *x* ^~^) = (*y*⇝*z*)→(*x*⇝*z*).

By (a) and (pR2), we have 1 = 1 → 1 ≤ (1⇝*x*)→(1⇝*x*) = *x* → *x*, and so *x* → *x* = 1. Similarly, *x*⇝*x* = 1.

If *x* ≤ *y*, by (pR5) and (b), we have *x* → *y* = *x* → *x*∨*y* = (*x* → *x*)∨(*x* → *y*) = 1. Conversely, if *x* → *y* = 1, by (pR2) and (a), we have *x* = 1⇝*x* ≤ (*x* → *y*)⇝(1 → *y*) = 1⇝*y* = *y*. Similarly, *x* ≤ *y* if and only if *x*⇝*y* = 1.

(pL2): by (c) and (pR1), *x* ≤ *y* if and only if *x* → *y* = 1 if and only if *y* ^−^⇝*x* ^−^ = 1 if and only if *y* ^−^ ≤ *x* ^−^. Similarly, *x* ≤ *y* if and only if *x*⇝*y* = 1 if and only if *y* ^~^ → *x* ^~^ = 1 if and only if *y* ^~^ ≤ *x* ^~^.

(pL3): since 0 ≤ 1^−^, by (pL2), 1^−~^ ≤ 0^~^. By (pL3′), 1 ≤ 0^~^; thus 1 = 0^~^ and 1^−^ = 0^~−^ = 0. Similarly, 1 = 0^−^ and 1^~^ = 0.

By (pR2) and (pR1), *x* = 1 → *x* = *x* ^−^⇝0, and so *x* ^−~^ = *x* ^−~−^⇝0 = 1 → *x* ^−~^ = *x* ^−^⇝0 = *x*. Hence, *x* ^−~^ = *x*. Similarly, we have *x* ^~−^ = *x*.

(pR4): by (pL2) and (pL3), it is easy to verify that pseudo-Kleene dual law holds:

(d) (*x*∧*y*)^~^ = *x* ^~^∨*y* ^~^, (*x*∨*y*)^~^ = *x* ^~^∧*y* ^~^, (*x*∧*y*)^−^ = *x* ^−^∨*y* ^−^, and (*x*∨*y*)^−^ = *x* ^−^∧*y* ^−^.

By (pR1), (pR5), and (d), *x*∧*y* → *z* = *z* ^−^⇝(*x*∧*y*)^−^ = *z* ^−^⇝*x* ^−^∨*y* ^−^ = (*z* ^−^⇝*x* ^−^)∨(*z* ^−^⇝*y* ^−^) = (*x* → *z*)∨(*y* → *z*). Similarly, we have *x*∧*y*⇝*z* = (*x*⇝*z*)∨(*y*⇝*z*).

If *x* ≤ *y*, then *y* → *z* ≤ (*x* → *z*)∨(*y* → *z*) = *x*∧*y* → *z* = *x* → *z* and *y*⇝*z* ≤ (*x*⇝*z*)∨(*y*⇝*z*) = *x*∧*y*⇝*z* = *x*⇝*z*.

Now we prove that (pR4) holds. Since *x* = 1 → *x* ≤ (*x* → *z*)⇝(1 → *z*) = (*x* → *z*)⇝*z*, *x* → (*y*⇝*z*)≥((*x* → *z*)⇝*z*)→(*y*⇝*z*) ≥ *y*⇝(*x* → *z*). Hence, *x* → (*y*⇝*z*) = *y*⇝(*x* → *z*).

**Corollary 9 .**

A structure (*A*, ∨, ∧, →, ⇝,  ^−^,  ^~^, 0,1) is a pseudo-*R*_0_ algebra if and only if it satisfies (pL1), (pL3′), (pR1), (pR2), (pR3), (pR5), and

(pR6) (*x* → *y*)∨((*x* → *y*)⇝(*x* ^−^∨*y*)) = (*x*⇝*y*)∨((*x*⇝*y*)→(*x* ^~^∨*y*)) = 1.

According to Theorem 8 and Corollary 9, one obtains most simplified definitions of pseudo-weak-*R*_0_ algebras and pseudo-*R*_0_ algebras, as the axiom systems are mutually independence (see Theorem 11).

**Definition 10 .**

A pseudo-weak-*R*_0_ algebra is a structure (*A*, ∧, ∨, →, ⇝,^−^,^~^, 0,1) such that (*A*, ∧, ∨, 0,1) is a bounded lattice and (1^~^)^−^ = (1^−^)^~^ = 1 and (0^~^)^−^ = (0^−^)^~^ = 0, satisfying the following axioms:

(P1) *x* → *y* = *y* ^−^⇝*x* ^−^, *x*⇝*y* = *y* ^~^ → *x* ^~^,
(P2) 1 → *x* = 1⇝*x* = *x*,
(P3) *x* → *y* ≤ (*z* → *x*)→(*z* → *y*), *x*⇝*y* ≤ (*z*⇝*x*)⇝(*z*⇝*y*),
(P4) *x* → (*y*∨*z*) = (*x* → *y*)∨(*x* → *z*), *x*⇝(*y*∨*z*) = (*x*⇝*y*)∨(*x*⇝*z*).

A pseudo-*R*_0_ algebra *A* is a pseudo-weak-*R*_0_ algebra satisfying the additional axiom as follows:

(P5) (*x* → *y*)∨((*x* → *y*)⇝(*x* ^−^∨*y*)) = (*x*⇝*y*)∨((*x*⇝*y*)→(*x* ^~^∨*y*)) = 1.

**Theorem 11 .**

The five axioms of Definition 10 are mutually independent.

**Proof**

Let *A* = [0,1], *x*∨*y* = max⁡{*x*, *y*}, *x*∧*y* = min⁡{*x*, *y*}, $x^{-}=\sqrt{1-x}$, and *x* ^~^ = 1 − *x* ^2^. Then *A* is a bounded lattice satisfying *x* ^−~^ = *x* ^~−^ = *x* for any *x* ∈ *A*.

(i) Define operations → and ⇝ as pseudo-Godel implication on *A* as follows:
  (2) $$\begin{array}{c} x⟶y=x⇝y=\{\begin{array}{cc} 1, & x\leq y,\\ y, & \text{otherwise}. \end{array} \end{array}$$

Then *A* satisfies (P2)–(P5), but not (P1): 1 → 0.5 = 0.5, but $0.5^{-}⇝1^{-}=\sqrt{0.5}⇝0=0$.

(ii) Define operations → and ⇝ on *A* as follows:
  (3) $$\begin{array}{c} x⟶y=x⇝y=1. \end{array}$$

Clearly, *A* satisfies (P1) and (P3)–(P5), but not (P2): 1 → 0.5 = 1⇝0.5 = 1 ≠ 0.5.

(iii) Define operations → and ⇝ on *A* as follows:
  (4) $$\begin{array}{c} x⟶y=\{\begin{array}{cc} x^{-}\vee y, & x=1\;\text{or}\;y=0,\\ 1, & \text{otherwise}, \end{array}\\ x⇝y=\{\begin{array}{cc} x^{\sim }\vee y, & x=1\;\text{or}\;y=0,\\ 1, & \text{otherwise}. \end{array} \end{array}$$

Then *A* satisfies (P1)-(P2) and (P4)-(P5), but not (P3). In fact, let *x* = 0.64, *y* = 0.1, and *z* = 0, then $y\to z=y^{-}\vee z=y^{-}=\sqrt{1-y}=\sqrt{0.9}\approx 0.95,(x\to y)\to (x\to z)=1\to x^{-}\vee z=1\to x^{-}=x^{-}=\sqrt{0.36}=0.6$.

(iv) Define operations → and ⇝ as pseudo-Lukasiewicz implication on *A* as follows:
  (5) $$\begin{array}{c} x⟶y=1\wedge \left(x^{-}+y\right),\;\;x⇝y=1\wedge \left(x^{\sim }+y\right). \end{array}$$

Then *A* satisfies (P1)–(P4), but not (P5): (0.19 → 0.07)∨[(0.19 → 0.07)⇝(0.19^−^∨0.07)] = 0.97∨(0.97⇝0.9) ≤ 0.97∨0.96 = 0.97 < 1.

(v) Suppose that *A* is a bounded lattice given by Figure 1.

The operations  ^−^,  ^~^, →, and ⇝ on *A* are defined by the following:

(6) x0fcdabe1x−=x~1ebadcf0x⟶y=x⇝y={1,x≤y,x−∨y=x~∨y,otherwise.

Then *A* satisfies (P1)–(P3) and (P5), but not (P4): (*a* → *b*)∨(*a* → *c*) = (*a* ^−^∨*b*)∨(*a* ^−^∨*c*) = (*d*∨*b*)∨(*d*∨*c*) = *b*∨*a* = *e*, but *a* → *b*∨*c* = *a* → *e* = 1.

## 4. Filters and Normal Filters of Pseudo-Weak-*R*_0_ Algebras

We introduce the notions of filters and normal filters in pseudo-weak-*R*_0_ algebras and investigate the structures and properties of the generated filters and generated normal filters in pseudo-weak-*R*_0_ algebras.

**Definition 12 .**

A nonempty subset *F* of a pseudo-weak-*R*_0_ algebra *A* is said to be a filter of *A* if it satisfies

(F1) *x*, *y* ∈ *F*⇒*x*⊙*y* ∈ *F*,
(F2) *x* ∈ *F*, *x* ≤ *y*⇒*y* ∈ *F*.

**Proposition 13 .**

For a subset *F* of a pseudo-weak-*R*_0_ algebra *A*, the following are equivalent:

- *F is a filter,*
- 1 ∈ *F and x*, *x*⇝*y* ∈ *F*⇒*y* ∈ *F*,
- 1 ∈ *F and x*, *x* → *y* ∈ *F*⇒*y* ∈ *F*.

**Proof**

(i)⇒(ii). By (F2), we have 1 ∈ *F*. By (F1), *x*, *x*⇝*y* ∈ *F*⇒*x*⊙(*x*⇝*y*) ∈ *F*. By (38) and (F2), *x*∧*y* ∈ *F*, and so *y* ∈ *F*.

(ii)⇒(iii). If *x*, *x* → *y* ∈ *F*, by (19), *x* ≤ (*x* → *y*)⇝*y*. By (4), *x*⇝((*x* → *y*)⇝*y*) = 1 ∈ *F*. By (ii), *y* ∈ *F*.

(iii)⇒(i). If *x* ∈ *F*, *x* ≤ *y*, then *x* → *y* = 1 ∈ *F*, so *y* ∈ *F*; that is, (F2) holds; if *x*, *y* ∈ *F*, by (41), *x* → (*y* → (*x*⊙*y*)) = (*x*⊙*y*)→(*x*⊙*y*) = 1 ∈ *F*, and so *x*⊙*y* ∈ *F*, which means (F1) holds.

Clearly, {1} and *A* are both filters of a pseudo-weak-*R*_0_ algebra *A*.

**Proposition 14 .**

*For a subset F* *of a pseudo-weak-R*_0_ *algebra A* *, the following are equivalent:*

- *F is a filter,*
- *x*, *y* ∈ *F*, *y* ≤ *x* → *z*⇒*z* ∈ *F*,
- *x*, *y* ∈ *F*, *y* ≤ *x*⇝*z*⇒*z* ∈ *F*.

**Proof**

(i)⇔(ii). If *x*, *y* ∈ *F*, *y* ≤ *x* → *z*, by (F2) and Proposition 13 (iii), *z* ∈ *F*. Conversely, if *x* ∈ *F*, by *x* ≤ *x* → 1, we have 1 ∈ *F*; suppose that *x*, *x* → *y* ∈ *F*, by *x* → *y* ≤ *x* → *y*, we have *y* ∈ *F*. By Proposition 13 (iii), *F* is a filter.

(i)⇔(iii). Similarly.

Next, we consider filter generated by a set. It is easy to verify that the intersection of filters of *A* is also a filter. If *S*⊆*A*, the least filter containing *S*; that is, the intersection of all filters of *A* containing *S* is called the filter generated by *S* and denoted by [*S*). If *S* = {*a*}, [{*a*}) is written [*a*). Clearly

(7) $$\begin{array}{c} \left[S\right)=\cap \left\{T\;|\;S\subseteq T\subseteq A,T\;\text{is}\;\text{a}\;\text{filter}\;\text{of}\;A\right\}. \end{array}$$

**Theorem 15 .**

Let *A* be a pseudo-weak-*R*_0_ algebra and let *S* be a nonempty subset of *A*. Then

(8) [S) ={x∈A ∣ there  are  n≥1, a1,a2,…,an∈S,     such  that  a1⊙⋯⊙an≤x} ={x∈A ∣ there  are  n≥1, a1,a2,…,an∈S,     such  that  an⇝(⋯⇝(a1⇝x)⋯)=1} ={x∈A ∣ there  are  n≥1, a1,a2,…,an∈S,     such  that  an⟶(⋯⟶(a1⟶x)⋯)=1}.

**Proof**

Only prove the first equality. Using (34) to the first equality, we can get the rest of the two equalities. Let *B* denote the right side of the first equality. If *x*, *y* ∈ *B*, then there are *a*_1_, *a*_2_,…, *a*_*n*_, *b*_1_, *b*_2_,…, *b*_*m*_ ∈ *S* such that *a*_1_⊙⋯⊙*a*_*n*_ ≤ *x* and *b*_1_⊙⋯⊙*b*_*m*_ ≤ *y*. By (37), *a*_1_⊙⋯⊙*a*_*n*_⊙*b*_1_⊙⋯⊙*b*_*m*_ ≤ *x*⊙*y*, so *x*⊙*y* ∈ *B*. If *x* ∈ *B* and *x* ≤ *y*, we have *a*_1_⊙⋯⊙*a*_*n*_ ≤ *x* ≤ *y*, so *y* ∈ *B*. Hence *B* is a filter. If *C* is a filter and *S*⊆*C*, for any *x* ∈ *B*, there are *a*_1_, *a*_2_,…, *a*_*n*_ ∈ *S* such that *a*_1_⊙⋯⊙*a*_*n*_ ≤ *x*. By (F2), *x* ∈ *C*, hence *B*⊆*C*.

For convenience, we shall write $a^{n}:=\overset{n}{\;\overset{︷}{a⊙\cdots ⊙a}}$ and *a* ^0^ : = 1; $a\overset{n}{⇝}x:=\overset{n}{\;\overset{︷}{a⇝(\cdots ⇝(a}}\;⇝x)\cdots )$ and $a\overset{0}{⇝}x:=x$; $a\overset{n}{\to }x:=\overset{n}{\;\overset{︷}{a\to (\cdots \to (a}}\to x)\cdots )$ and $a\overset{0}{\to }x:=x$.

**Corollary 16 .**

If *A* is a pseudo-weak-*R*_0_ algebra and *a* ∈ *A*, then

(9) [a)={x∈A ∣ n≥1,an≤x}={x∈A ∣ n≥1,a⇝nx=1}={x∈A ∣ n≥1,a→nx=1}.

**Corollary 17 .**

*Let F* *be a filter of a pseudo-weak-R*_0_ *algebra A* *and a* ∈ *A; then*

(10) [F∪{a})={x∈A ∣ (s1⊙an1)⊙⋯⊙(sm⊙anm)≤x,     where  m≥1,n1,…,nm≥0,     s1,…,sm∈F}.

**Theorem 18 .**

Let *F* be a filter of a pseudo-weak-*R*_0_ algebra *A* and *a*, *b* ∈ *A*; then

(11) $$\begin{array}{c} \left[F\cup \left\{a\right\}\right)\cap \left[F\cup \left\{b\right\}\right)=\left[F\cup \left\{a\vee b\right\}\right). \end{array}$$

**Proof**

Assume that *x* ∈ [*F* ∪ {*a*})∩[*F* ∪ {*b*}), by Corollary 17, there are *n*_1_,…, *n*_*m*_, *l*_1_,…, *l*_*k*_ ≥ 0, *s*_1_,…, *s*_*m*_, *t*_1_,…, *t*_*k*_ ∈ *F* such that

(12) $$\begin{array}{c} \left(s_{1}⊙a^{n_{1}}\right)⊙\cdots ⊙\left(s_{m}⊙a^{n_{m}}\right)\leq x,\\ \left(t_{1}⊙b^{l_{1}}\right)⊙\cdots ⊙\left(t_{k}⊙b^{l_{k}}\right)\leq x. \end{array}$$

Put *p* = *s*_1_⊙⋯⊙*s*_*m*_⊙*t*_1_⊙⋯⊙*t*_*k*_ and *q* = max⁡{*n*_1_,…, *n*_*m*_, *l*_1_,…, *l*_*k*_}, and then

(13) $$\begin{array}{c} {\left(p⊙a^{q}\right)}^{m}\leq x,\;\;{\left(p⊙b^{q}\right)}^{k}\leq x. \end{array}$$

Thus, by (46) *x* ≥ (*p*⊙*a* ^*q*^)^*m*^∨(*p*⊙*b* ^*q*^)^*k*^ ≥ ((*p*⊙*a* ^*q*^)^*m*^∨(*p*⊙*b* ^*q*^))^*k*^ ≥ ((*p*⊙*a* ^*q*^)∨(*p*⊙*b* ^*q*^))^*mk*^ = (*p*⊙(*a* ^*q*^∨*b* ^*q*^))^*mk*^ ≥ (*p*⊙(*a*∨*b*)^*q*^2^^)^*mk*^. *x* ∈ [*F* ∪ {*a*∨*b*}). Hence [*F* ∪ {*a*})∩[*F* ∪ {*b*})⊆[*F* ∪ {*a*∨*b*}). Inverse contains is obvious.

**Corollary 19 .**

Let *F* be a filter of a pseudo-weak-*R*_0_ algebra *A* and *a*, *b* ∈ *A*. If *a*∨*b* ∈ *F*, then

(14) $$\begin{array}{c} \left[F\cup \left\{a\right\}\right)\cap \left[F\cup \left\{b\right\}\right)=F. \end{array}$$

**Corollary 20 .**

Let *A* be a pseudo-weak-*R*_0_ algebra and *a*, *b* ∈ *A*; then [*a*)∩[*b*) = [*a*∨*b*).

**Proof**

Taking *F* = {1} in Theorem 18.

Next we introduce the notion of normal filters in a pseudo-weak-*R*_0_ algebra.

**Definition 21 .**

A filter *F* of a pseudo-weak-*R*_0_ algebra *A* is called normal if *x*, *y* ∈ *A*, *x* → *y* ∈ *F* if and only if *x*⇝*y* ∈ *F*.

**Proposition 22 .**

Let *F* be a normal filter of a pseudo-weak-*R*_0_ algebra *A*. Then there is *s* ∈ *F* such that *b*⊙*s* ≤ *c* if and only if there is *t* ∈ *F* such that *t*⊙*b* ≤ *c*.

**Proof**

If there is *s* ∈ *F* such that *b*⊙*s* ≤ *c*, by (34), *s* ≤ *b*⇝*c*. By *s* ∈ *F*, we have *b*⇝*c* ∈ *F*, and so *b* → *c* ∈ *F*. Put *b* → *c* = *t* ∈ *F*, and then *t*⊙*b* ≤ *c*. Converse is similar.

**Theorem 23 .**

*If F* *is a normal filter of a pseudo-weak-R*_0_ *algebra A* *and a* ∈ *A, then*

(15) [F∪{a})={x∈A ∣ there  are  s∈F,n≥0,     such  that  s⊙an≤x}={x∈A ∣ there  are  s∈F,n≥0,     such  that  an⊙s≤x}.

**Proof**

We show the first equality. By Corollary 17,

(16) [F∪{a})={x∈A ∣ (s1⊙an1)⊙⋯⊙(sm⊙anm)≤x,     m≥1,n1,…,nm≥0s1,…,sm∈F}.

Since

(17) $$\begin{array}{c} \left(s_{1}⊙a^{n_{1}}\right)⊙\left(s_{2}⊙a^{n_{2}}\right)⊙\cdots ⊙\left(s_{m}⊙a^{n_{m}}\right)\leq x, \end{array}$$

by (34),

(18) $$\begin{array}{c} \left(s_{1}⊙a^{n_{1}}\right)⊙s_{2}\leq \left(a^{n_{2}}⊙\left(s_{3}⊙a^{n_{3}}\right)⊙\cdots ⊙\left(s_{m}⊙a^{n_{m}}\right)\right)⟶x, \end{array}$$

by Proposition 22, there is *t*_2_ ∈ *F* such that

(19) $$\begin{array}{c} t_{2}⊙\left(s_{1}⊙a^{n_{1}}\right)\leq \left(a^{n_{2}}⊙\left(s_{3}⊙a^{n_{3}}\right)⊙\cdots ⊙\left(s_{m}⊙a^{n_{m}}\right)\right)⟶x, \end{array}$$

and so

(20) $$\begin{array}{c} \left(t_{2}⊙s_{1}\right)⊙a^{n_{1}+n_{2}}⊙\left(s_{3}⊙a^{n_{3}}\right)⊙\cdots ⊙\left(s_{m}⊙a^{n_{m}}\right)\leq x. \end{array}$$

Repeating the above steps, there are *t*_2_,…, *t*_*m*_ ∈ *F* such that

(21) $$\begin{array}{c} \left(t_{m}⊙\cdots ⊙t_{2}⊙s_{1}\right)⊙a^{n_{1}+\cdots +n_{m}}\leq x. \end{array}$$

Let *s* = *t*_*m*_ + ⋯+*t*_2_ + *s*_1_ ∈ *F* and *n* = *n*_1_ + ⋯+*n*_*m*_, we have *s*⊙*a* ^*n*^ ≤ *x*. That is that the first equality holds.

By the first equality and Proposition 22, we can obtain the second equation.

**Corollary 24 .**

*If F* *is a normal filter of a pseudo-weak-R*_0_ *algebra A* *and a* ∈ *A, then*

(22) [F∪{a})={x∈A ∣ there  is  n≥0,such  that  a→nx∈F}={x∈A ∣ there  is  n≥0,such  that  a⇝nx∈F}.

**Proof**

By Theorem 23,

(23) [F∪{a})={x∈A ∣ there  are  s∈F,n≥0,     such  that  s⊙an≤x}.

Since there is *s* ∈ *F* such that *s*⊙*a* ^*n*^ ≤ *x*, if and only if there is *s* ∈ *F* such that $s\leq \overset{n}{\;\overset{︷}{a\to (\cdots \to (a}}\to x)\cdots )$; that is, there is *s* ∈ *F* such that $s\leq a\overset{n}{\to }x$, if and only if $a\overset{n}{\to }x\in F$. Thus, we prove the first equality.

Similarly, by

(24) [F∪{a})={x∈A ∣ there  are  s∈F,n≥0,     such  that  an⊙s≤x}.

Since there is *s* ∈ *F* such that *a* ^*n*^⊙*s* ≤ *x*, if and only if there is *s* ∈ *F* such that $s\leq \overset{n}{\;\overset{︷}{a⇝(\cdots ⇝(a}}⇝x)\cdots )$; that is, there is *s* ∈ *F* such that $s\leq a\overset{n}{⇝}x$, if and only if $a\overset{n}{⇝}x\in F$, Thus, we have the second equality.

**Corollary 25 .**

*If F* *is a normal filter of a pseudo-weak-R*_0_ *algebra A* *and a* ∈ *A, then*

(25) [F∪{a})={x∈A ∣ there  is  n≥0,such  that  an⟶x∈F}={x∈A ∣ there  is  n≥0,such  that  an⇝x∈F}.

**Proof**

There is *s* ∈ *F* such that *s*⊙*a* ^*n*^ ≤ *x*, if and only if there is *s* ∈ *F* such that *s* ≤ *a* ^*n*^ → *x*, if and only if *a* ^*n*^ → *x* ∈ *F*. There is *s* ∈ *F* such that *a* ^*n*^⊙*s* ≤ *x*, if and only if there is *s* ∈ *F* such that *s* ≤ *a* ^*n*^⇝*x*, if and only if *a* ^*n*^⇝*x* ∈ *F*. By Theorem 23, Corollary 25 holds.

## 5. Conclusions

We obtained the most simplified axiom systems of pseudo-weak-*R*_0_ algebras and pseudo-*R*_0_ algebras and proved the mutually independence of axioms. We introduced the notions of filters and normal filters in pseudo-weak-*R*_0_ algebras and gave the structures and properties of the generated filters and generated normal filters in pseudo-weak-*R*_0_ algebras. These will be conducive to further study pseudo-weak-*R*_0_ algebras (pseudo-IMTL algebras) and pseudo-*R*_0_ algebras (pseudo-NM algebras). In the future, we will investigate relations between various kinds of filters of pseudo-logic algebras. We may also study fuzzy type of filters of pseudo-weak-*R*_0_ algebras and pseudo-*R*_0_ algebras.

## Figures and Tables

**Figure 1:**